# Selection and maintenance of mobile linezolid-resistance genes and plasmids carrying them in the presence of florfenicol, an animal-specific antimicrobial

**DOI:** 10.1099/acmi.0.000997.v3

**Published:** 2025-04-08

**Authors:** Akira Fukuda, Masaru Usui

**Affiliations:** 1 Laboratory of Food Microbiology and Food Safety, Department of Health and Environmental Sciences, School of Veterinary Medicine, Rakuno Gakuen University, Ebetsu, 069-8501, Hokkaido, Japan

**Keywords:** antimicrobial resistance, florfenicol, linezolid, mobile antimicrobial resistance gene

## Abstract

Mobile linezolid-resistance genes (*optrA*, *poxtA* and *cfr*) that confer resistance to linezolid and florfenicol have been detected globally in various sources. Linezolid is a last-resort antimicrobial used in human clinical settings, and florfenicol is commonly used in veterinary clinical settings. The present study sought to evaluate the potential of florfenicol in veterinary use to select for linezolid-resistant bacteria. The growth and fitness of linezolid-resistant bacteria harbouring mobile linezolid-resistance genes were assessed in the presence and absence of florfenicol using *Enterococcus faecalis* and *Enterococcus faecium*, respectively. The bacterial strains harboured wild and cloning plasmids carrying mobile linezolid-resistance genes, which reduced their susceptibility to linezolid and florfenicol. The acquisition of plasmids carrying mobile linezolid-resistance genes improved bacterial growth in the presence of florfenicol and conferred fitness costs in its absence. Florfenicol imposes a selection pressure on bacteria harbouring plasmids carrying mobile linezolid-resistance genes. Hence, the appropriate use of florfenicol in veterinary clinical settings is important to control the dissemination of mobile linezolid-resistance genes and to ensure the sustained effectiveness of linezolid against multidrug-resistant bacteria, including vancomycin-resistant enterococci in human clinical settings.

## Data Summary

The authors confirm that all supporting data have been provided within the article as figures and tables or through Excel file with the article.

## Introduction

Antimicrobial resistance is a global concern, and antimicrobial-resistant bacteria have emerged and been selected using antimicrobials in human and veterinary clinical settings [1]. Oxazolidinones, such as linezolid and tedizolid, are approved only for clinical use in humans. They are one of the last resort antimicrobials for the treatment of clinically important antimicrobial-resistant Gram-positive bacteria including vancomycin-resistant enterococci and methicillin-resistant *Staphylococcus aureus* [2]. However, oxazolidinone-resistant bacteria have been isolated from humans, livestock, food and environment including fields where oxazolidinones are not used [3].

Mechanisms underlying the attainment of linezolid resistance are acquisition of mobile linezolid-resistance genes (*optrA*, *poxtA* and *cfr*) and chromosomal mutations in 23S rRNA and ribosomal protein L3 and L4 [4,5]. In the absence of antimicrobials, mobile linezolid-resistance genes confer a lower fitness cost to the host bacteria than 23S rRNA mutations [6,7]. The transmission of mobile linezolid-resistance genes across bacterial species by mobile genetic elements, including plasmids and insertion sequences, limits the available therapeutic options, thereby resulting in a serious impact on public health [4].

Mobile linezolid-resistance genes reduce the susceptibility to linezolid and other types of antimicrobials such as phenicols via ribosomal protection by *OptrA* and *PoxtA*, as well as methylation of rRNA-binding sites by *Cfr* [3]. Florfenicol is a bacteriostatic antimicrobial, and the use of florfenicol in livestock and aquaculture has increased over the past few decades [8]. Florfenicol use may be related to the selection of bacteria harbouring mobile linezolid-resistance genes [9]. Moreover, plasmids carrying mobile linezolid-resistance genes often co-harbour florfenicol-resistance genes, especially *fexA* and *fexB* as efflux proteins [10]. However, further evaluation is needed to understand the effect of mobile linezolid-resistance genes and plasmids carrying them on bacterial growth and fitness in the presence and absence of florfenicol [7,11,14]. Thus, elucidating the selective pressure on mobile linezolid-resistance genes and their plasmids in livestock is important.

This study aimed to elucidate the selection pressure of florfenicol use on linezolid-resistance genes and their plasmids. We evaluated the bacterial growth and fitness of linezolid-resistant bacteria harbouring mobile linezolid-resistance genes in the presence and absence of florfenicol.

## Methods

### Bacterial strains

In our previous study, plasmid-mediated *optrA*- and/or *poxtA*-positive enterococci isolates (K6_D, L6_D and L6_E) were isolated transformants of FA2-2, and transconjugants of BM4105RF were made by transformation and conjugation using isolates and recipients (Table 1) [5]. These transformants and transconjugants of enterococci that acquired the *optrA*- and/or *poxtA*-carrying plasmids were used in this study. The antimicrobial susceptibility and antimicrobial resistance genes have been reported. The *optrA*-carrying plasmid L6D_p1 and *poxtA*-carrying plasmids K6D_p2 and L6E_p1 co-harboured the *fexA* or *fexB* genes. Transformants TF_K6D_FA, TF_L6D_FA and TF_L6E_FA were the parental strains from which *Enterococcus faecalis* FA2-2 acquired the *optrA*- or *poxtA*-carrying plasmids derived from the K6D, L6D and L6E strains, respectively. Transconjugants of TC_K6D_FA were a parental strain from which *Enterococcus faecium* BM4105RF acquired the *optrA*- and *poxtA*-carrying plasmids derived from the K6D strain.

**Table 1.** Enterococci strains used in this study

Strains	Name	Species	Minimum inhibitory concn (μg/ml)			Linezolid-resistance genes carrying plasmids
			Linezolid	Florfenicol	Chloram-phenicol	
Isolates	K6_D	*E. faecium*	**8**	**64**	**32**	K6D_p1: *optrA, ermA, ermB, aac(6')-aph(2''), ant(6)-Ia, aph(3')-III, dfrG, lsa(E*)
						K6D_p2: *poxtA, fexB, ermB, tet(L*)
	L6_D	*E. faecalis*	**4**	**64**	**64**	L6D_p1: *optrA, fexA, ermA*
	L6_E	*E. hirae*	**4**	**32**	**16**	L6E_p1: *poxtA, fexB, aph(3’)-III, tet(L), tet(M*)
Recipient	FA2-2	*E. faecalis*	2	4	4	
	BM4105RF	*E. faecium*	2	4	4	
Transformants of FA2-2	TF_K6D_FA	*E. faecalis*	**4**	**32**	**16**	K6D_p2
	TF_L6D_FA	*E. faecalis*	**8**	**64**	**32**	L6D_p1
	TF_L6E_FA	*E. faecalis*	**8**	**32**	**16**	L6E_p1
Transconjugants of BM4105RF	TC_K6D_BM	*E. faecium*	**8**	**16**	**32**	K6D_p1, K6D_p2
Cloning strains carrying pAM401	pAM_FA	*E. faecalis*	2	2	128*	
	pAM_BM	*E. faecium*	2	2	32*	
	pAM-optrA_FA	*E. faecalis*	**8**	**32**	128*	pAM401: *optrA*
	pAM-optrA_BM	*E. faecium*	**8**	**64**	32*	pAM401: *optrA*
	pAM-poxtA_FA	*E. faecalis*	**4**	**64**	64*	pAM401: *poxtA*
	pAM-poxtA_BM	*E. faecium*	**4**	8	32*	pAM401: *poxtA*
	pAM-cfr_FA	*E. faecalis*	**4**	**64**	128*	pAM401: *cfr*
	pAM-cfr_BM	*E. faecium*	**4**	**32**	32*	pAM401: *cfr*

Resistance is indicated in bold and underlined font. Intermediate is indicated in bold font

*The shuttle vector pAM401 carries a pIP501-analogous *cat* gene that confers high-level resistance to chloramphenicol, but not to florfenicol.

### Cloning of transferable linezolid-resistance genes

The *optrA*- and *poxtA*-carrying plasmids used in this study co-carried the *fexA* or *fexB* genes. To evaluate the bacterial growth and fitness of linezolid-resistant bacteria harbouring mobile linezolid-resistance genes in the presence and absence of florfenicol, only mobile linezolid-resistance gene-carrying plasmids were constructed. To clone *optrA* and *poxtA* genes of plasmids in *E. faecium* K6D (accession no. SAMD00577372) and *cfr* gene of *S. lentus* HB162 (accession no. SAMD00827542) from laboratory stock strains, DNA segments were amplified by PCR using specific primers (Table S1, available in the online version of this article) [5,13,16]. PCR amplicons of *optrA* and *poxtA* genes were digested with *Bam*HI and *Xba*I (Takara Bio Inc., Tokyo, Japan), and that of *cfr* gene amplicon was digested with *Bam*HI and *Sal*I (Takara Bio Inc.). These digested fragments were ligated into plasmid pAM401 (ATCC37429) using the TaKaRa DNA Ligation Kit LONG (Takara Bio Inc.), and ligated plasmids were transformed into *Escherichia coli* DH5α cells. The ligated plasmids extracted from *E. coli* DH5α were transformed into *E. faecalis* FA2-2 and *E. faecium* BM4105RF (Table 1), as described previously [5,17]. To prepare electrocompetent of *E. faecalis* FA2-2 and *E. faecium* BM4105RF cells, overnight cultures of recipient strains were inoculated in M17 broth (BD Biosciences) supplemented with 0.5 M sucrose and glycine (8% for *E. faecalis* FA2-2 and 3% for *E. faecium* BM4105RF) and incubated at 37 °C for 12–24 h. The cells were subsequently washed thrice with ice-cold electroporation buffer (0.5 M sucrose and 10% glycerol), and the cell suspensions were used for electroporation. The extracted plasmids were electroporated into recipient strains treated with glycine using the ECM 630 Exponential Decay Wave Electroporation System (BTX, San Diego, CA, USA) under the following conditions: voltage, 12.5 kV/cm; capacitance, 25 µF; and resistance, 200 Ω.

### Susceptibility testing

The susceptibility to linezolid, florfenicol and chloramphenicol (Tokyo Chemical Industry Co., Ltd., Tokyo, Japan) was determined using the microbroth dilution method in accordance with the Clinical Laboratory Standards Institute (CLSI) guidelines [18]. The breakpoints of linezolid and chloramphenicol were defined in the CLSI. The epidemiological cut-off values of florfenicol in MIC EUCAST (https://mic.eucast.org/) were applied to *E. faecium* and *E. faecalis*, and a minimum inhibitory concentration of >8 µg ml^−1^ was considered as resistant to florfenicol. *E. faecalis* ATCC29212 and *S. aureus* ATCC29213 were used as quality control strains.

### Microbial growth in the presence or absence of florfenicol

The growth of the transformants and transconjugants with the acquired mobile linezolid-resistance gene-carrying plasmids was compared with their parental recipient strains and the cloning strains carrying the mobile linezolid-resistance gene-carrying pAM401 plasmids and empty pAM401 plasmid in the presence or absence of florfenicol [19]. The tested enterococci strains were incubated in Mueller-Hinton broth (MHB) (Oxoid Ltd, Hampshire, UK) at 37 ℃ overnight. The cultures were sub-cultured (1 : 200) in fresh MHB with or without florfenicol (4 and 32 µg ml^−1^), followed by their addition to individual wells of a 96-well flat-bottom plate. The plate was incubated at 37 ℃ for 16 h with orbital shaking, and OD_600_ was measured every 15 min using a Nivo^TM^ multimode plate reader (PerkinElmer, Inc., Waltham, MA, USA). The test was performed twice with three biologically independent replicates.

### Pairwise competition assay

As previously described [20,21], overnight MHB cultures of pair strains [transformants, transconjugants and their recipient strains; mobile linezolid-resistance gene-cloning and their empty vector (pAM401) strains] were mixed in a 1 : 1 ratio (5 µl each) in 10 ml of fresh MHB. The mixtures were incubated at 37 °C for 24 h. Thereafter, 10 µl of the overnight culture was added to 10 ml of fresh MHB. After sub-culturing for 10 days, the cultures were taken out and subjected to 10-fold serial dilutions. The diluents were plated on MHA plates with or without florfenicol (4 µg ml^−1^) to calculate the number of cells for each strain. Relative fitness was calculated as follows: w=ln (NRt/NR0)/ln (NSt/NS0). NR: number of resistant strains harbouring the plasmid-mediated mobile linezolid-resistance genes (transformants, transconjugants and mobile linezolid-resistance gene-cloning strains); NS: number of susceptible strains (recipient strains and empty vector strains). A value below one indicates the existence of a fitness cost.

## Results and discussion

### Antimicrobial susceptibility of mobile linezolid-resistance gene cloning strains of enterococci

The expression of *optrA*, *poxtA* and *cfr* genes from the cloned DNA resulted in reduced linezolid and florfenicol susceptibility of the linezolid-resistance gene cloning strains of *E. faecalis* and *E. faecium* (Table 1). The chloramphenicol-resistance gene *cat* in pAM401 did not affect the susceptibility to linezolid and florfenicol. Acquisition of *optrA*, *poxtA* and *cfr* genes reduces the susceptibility of enterococci and/or staphylococci to linezolid and florfenicol [13,15]. Plasmid-mediated mobile linezolid-resistance genes have been reported in several bacterial species, including human and animal pathogens [3]. It is important to study the prevalence of mobile linezolid-resistance genes and the bacterial species harbouring them in order to prevent the dissemination and circulation of antimicrobial resistance [22]. These results demonstrate the importance of mapping the prevalence of antimicrobial resistance genes in multiple bacterial species, which would provide accurate information regarding the prevalence of bacteria harbouring mobile linezolid-resistance genes.

### Bacterial growth in the presence or absence of florfenicol

**Fig. 1.**
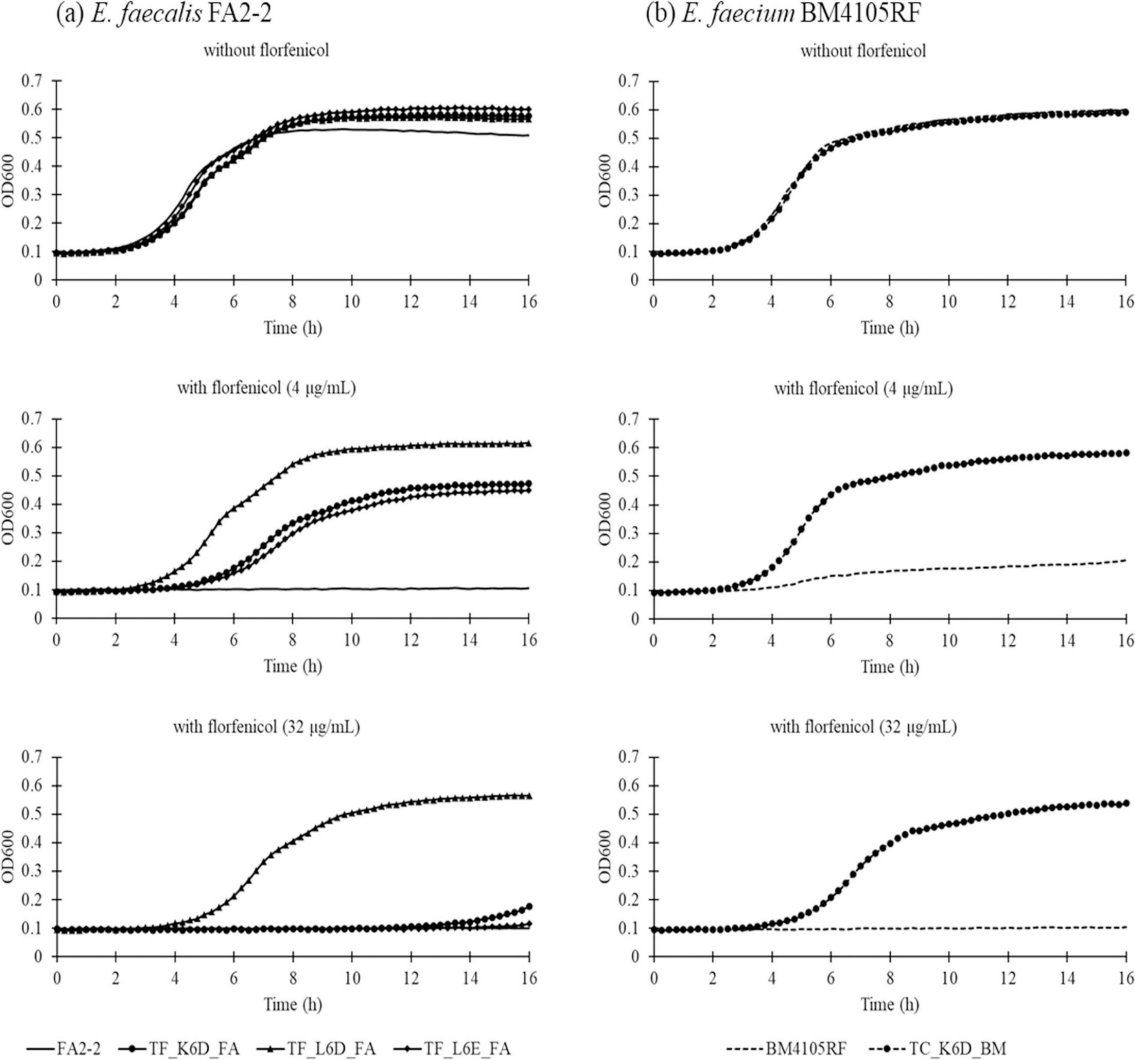
Growth rate of (**a**) transformants of *E. faecalis* FA2-2 and (**b**) transconjugants of *E. faecium* BM4105RF harbouring wild plasmids carrying mobile linezolid-resistance genes in the presence and absence of florfenicol.

**Fig. 2.**
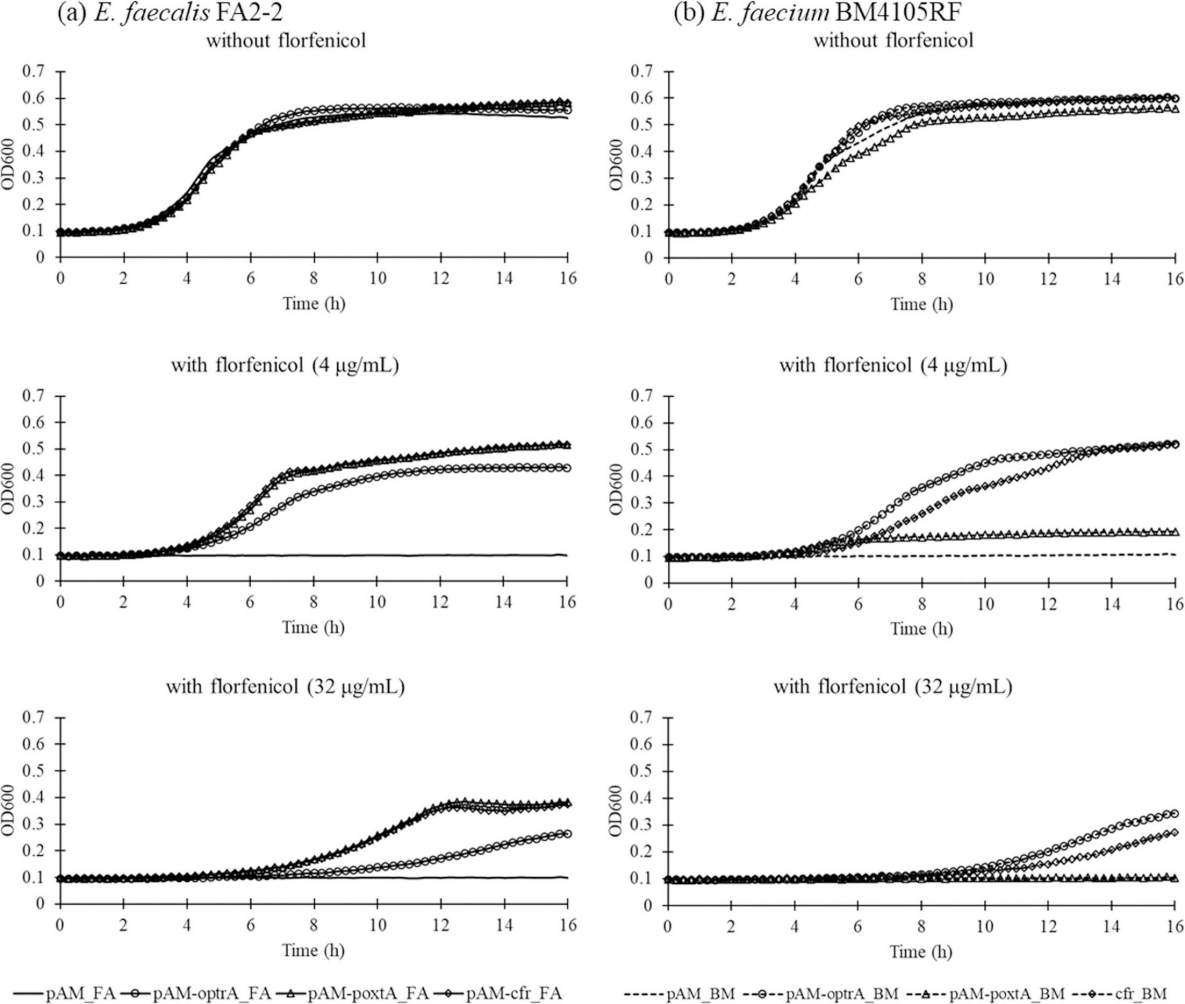
Growth rate of (**a**) *E. faecalis* FA2-2 and (**b**) *E. faecium* BM4105RF harbouring pAM401 plasmids carrying mobile linezolid-resistance genes in the presence and absence of florfenicol.

The bacterial growth of the transformants and transconjugants having linezolid-resistance genes on plasmids and their recipient strains of *E. faecalis* FA2-2 and *E. faecium* BM4105RF was evaluated in the presence or absence of florfenicol (Fig. 1). In the absence of florfenicol, the bacterial growth of transformants, transconjugants and their recipients was almost similar. In the presence of 4 µg ml^−1^ florfenicol, TF_L6D_FA showed better growth than TF_K6D_FA and TF_L6E_FA, while *E. faecalis* FA2-2 did not show any growth (Fig. 1a). In the presence of 32 µg ml^−1^ florfenicol, only TF_L6D_FA showed growth, while the other transformants and *E. faecalis* FA2-2 did not. With 4 and 32 µg ml^−1^ florfenicol, TC_K6D_BM showed growth, while *E. faecium* BM4105RF showed negligible growth (Fig. 1b). The bacterial growth of the cloning strains carrying cloned linezolid-resistance genes on plasmid pAM401 and empty vector was studied in the presence or absence of florfenicol (Fig. 2). The absence of florfenicol, the bacterial growth of strains carrying cloning and empty vector plasmids of pAM401 was almost similar in the absence of florfenicol. In the presence of 4 and 32 µg ml^−1^ florfenicol, the cloning *E. faecalis* FA2-2 strains, pAM-poxtA_FA and pAM-cfr_FA, showed better growth than pAM-optrA_FA, while pAM_FA did not show any growth (Fig. 2a). Among the cloning strains of *E. faecium* BM4105RF, pAM-optrA_BM and pAM-cfr_BM showed growth, while pAM_poxtA_BM had negligible growth (Fig. 2b). These results indicate a preferential selection of bacteria harbouring linezolid-resistance genes on plasmids in the presence of florfenicol. Previous studies have reported an association between residual florfenicol and the abundance of mobile linezolid-resistance genes in livestock manure [9]. Plasmids carrying mobile linezolid-resistance genes often co-harbour phenicol-resistance genes, including *fexA* and *fexB*; bacteria harbouring these plasmids get preferentially selected in the presence of florfenicol [5,11]. A previous report has shown that, in the presence of linezolid, an *E. faecium* strain carrying plasmid-mediated *poxtA* exhibited enhanced resistance to oxazolidines and phenicols through *poxtA* amplification [12]. Therefore, a clear understanding of the dynamics and evolution paths of bacteria harbouring mobile linezolid-resistance genes in the presence of antimicrobials is important to ensure the appropriate use of florfenicol and linezolid in veterinary and human clinical settings, respectively.

### Pairwise competition assay

**Fig. 3.**
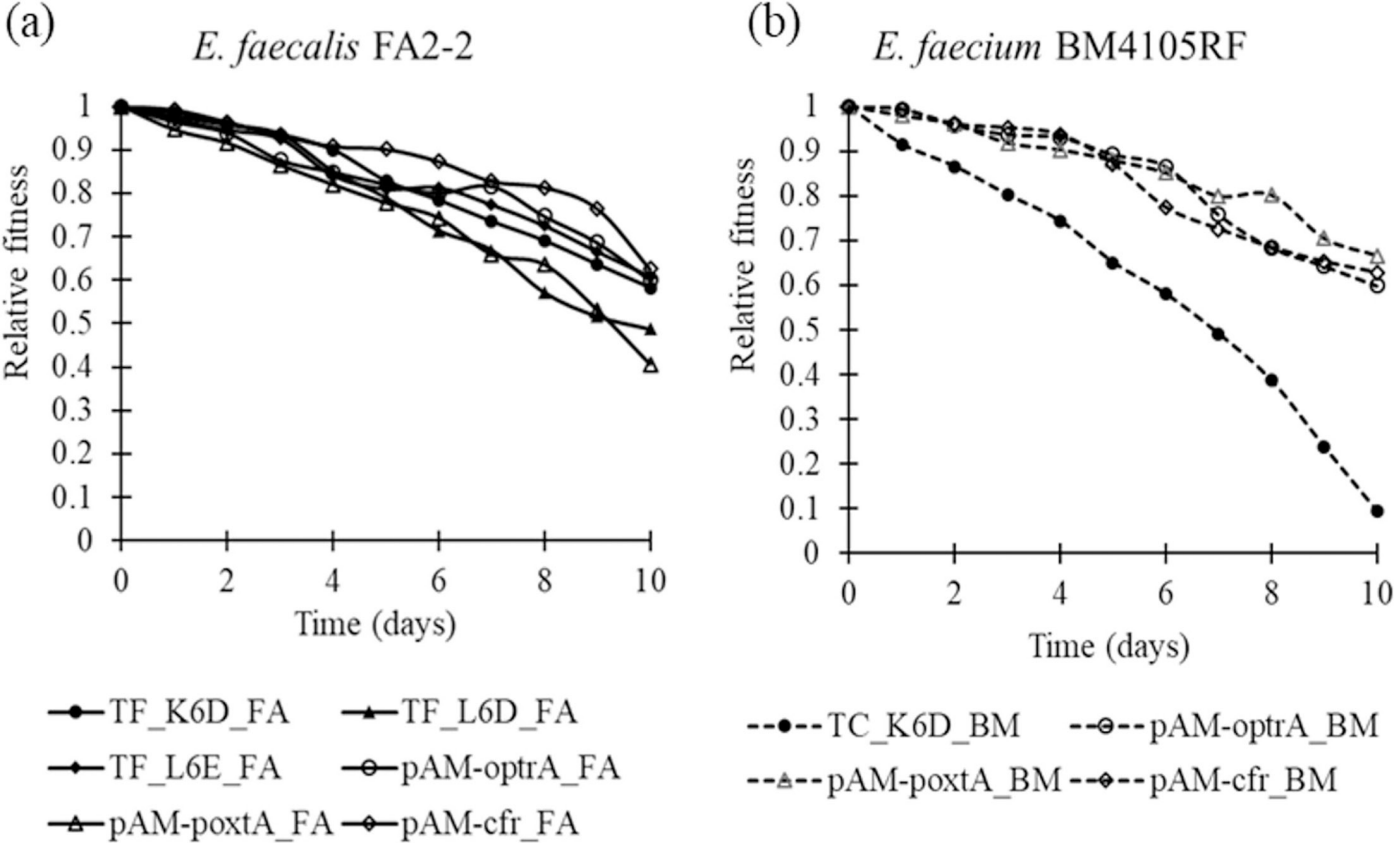
Fitness cost of plasmids carrying mobile linezolid-resistance genes on (**a**) *E. faecalis* FA2-2 and (**b**) *E. faecium* BM4105RF in the absence of florfenicol.

In the absence of florfenicol, values of relative fitness were less than one for all pairs (Fig. 3). Acquisition of mobile linezolid-resistance genes on wild and pAM401 plasmids conferred a fitness cost on the growth of *E. faecalis* FA2-2 and *E. faecium* BM4105RF. The fitness cost of mobile linezolid-resistance genes and plasmids carrying them is lower than that of chromosomal mutations [4,7, 12]. The chromosomal mutations conferring linezolid resistance were stable in bacteria as hospital-adapted clones; however, in a few cases, plasmid-mediated linezolid-resistance genes were unstable due to plasmid curing [11,23]. The wild plasmids used in this study were not cured in the absence of antimicrobials and could be transferred; however, they conferred a fitness cost to the host bacteria [5]. Accessory genes in plasmids carrying antimicrobial resistance genes may have beneficial effects on bacterial growth in the absence of antimicrobials [6,21]. The plasmids evolved to adapt to host bacteria and growth conditions [24]. The dissemination of plasmids carrying antimicrobial resistance genes is a major public health concern, and further research is required regarding the maintenance factors and mechanisms of plasmids carrying antimicrobial resistance genes.

In *E. faecium* BM4105RF, TC_K6D_BM, which harbours two *optrA-* and *poxtA*-carrying plasmids, exhibited a higher fitness cost than the other strains (Fig. 3b). These plasmids, alongside other similar ones, were detected in clade A2 *E. faecium* ST324 in Japan and France [5], which could indicate simultaneous adaptive evolution of the plasmids and host bacteria [25,26]. The clade A2 of *E. faecium* causes sporadic infections in animals and humans [27]. In particular, the presence of multidrug-resistant and high-virulence lineages harbouring plasmids with multidrug-resistance genes is highly concerning due to limited antimicrobial therapeutic options in clinical situations [28]. Hence, it is crucial to analyse the status of specific and pandemic lineages of bacterial clones and plasmids to understand the trends in the dissemination and evolution of antimicrobial-resistant strains.

## Conclusions

The presence of florfenicol is advantageous for the growth of bacteria harbouring plasmid-borne linezolid-resistance genes; however, the latter confer a fitness cost to bacteria in the absence of the antimicrobial. Bacteria harbouring mobile linezolid-resistance genes could be selected and maintained in livestock using florfenicol, but not linezolid. Hence, the appropriate and limited use of antimicrobials in livestock is important for controlling the spread of mobile linezolid-resistance genes.

## Supplementary material

10.1099/acmi.0.000997.v3 Uncited Table S1.

## Abbreviations

CLSI: Clinical Laboratory Standards Institute
MHB: Mueller-Hinton broth

## References

[1] Woolhouse M, Ward M, van Bunnik B, Farrar J (2015). Antimicrobial resistance in humans, livestock and the wider environment. Phil Trans R Soc B.

[2] Bender JK, Cattoir V, Hegstad K, Sadowy E, Coque TM (2018). Update on prevalence and mechanisms of resistance to linezolid, tigecycline and daptomycin in enterococci in Europe: Towards a common nomenclature. Drug Resist Updat.

[3] Schwarz S, Zhang W, Du X-D, Krüger H, Feßler AT (2021). Mobile oxazolidinone resistance genes in gram-positive and gram-negative bacteria. Clin Microbiol Rev.

[4] Brenciani A, Morroni G, Schwarz S, Giovanetti E (2022). Oxazolidinones: mechanisms of resistance and mobile genetic elements involved. J Antimicrob Chemother.

[5] Fukuda A, Nakajima C, Suzuki Y, Usui M (2024). Transferable linezolid resistance genes (*optrA* and *poxtA*) in enterococci derived from livestock compost at Japanese farms. J Glob Antimicrob Resist.

[6] Liu Z, Zhao Q, Xu C, Song H (2024). Compensatory evolution of chromosomes and plasmids counteracts the plasmid fitness cost. Ecol Evol.

[7] Long KS, Vester B (2012). Resistance to linezolid caused by modifications at its binding site on the ribosome. Antimicrob Agents Chemother.

[8] Guo X, Chen H, Tong Y, Wu X, Tang C (2024). A review on the antibiotic florfenicol: occurrence, environmental fate, effects, and health risks. Environ Res.

[9] Wang Y, Li X, Fu Y, Chen Y, Wang Y (2020). Association of florfenicol residues with the abundance of oxazolidinone resistance genes in livestock manures. J Hazard Mater.

[10] Turner AM, Lee JYH, Gorrie CL, Howden BP, Carter GP (2021). Genomic insights into last-line antimicrobial resistance in multidrug-resistant *Staphylococcus* and vancomycin-resistant *Enterococcus*. Front Microbiol.

[11] Zhang E, Zong S, Zhou W, Zhou J, Han J (2022). Characterization and comparative genomics analysis of RepA_N multi-resistance plasmids carrying *optrA* from *Enterococcus faecalis*. Front Microbiol.

[12] Shan X, Li C, Zhang L, Zou C, Yu R (2024). *poxtA* amplification and mutations in 23S rRNA confer enhanced linezolid resistance in *Enterococcus faecalis*. J Antimicrob Chemother.

[13] Wang Y, Lv Y, Cai J, Schwarz S, Cui L (2015). A novel gene, optrA, that confers transferable resistance to oxazolidinones and phenicols and its presence in *Enterococcus faecalis* and *Enterococcus faecium* of human and animal origin. J Antimicrob Chemother.

[14] Antonelli A, D’Andrea MM, Brenciani A, Galeotti CL, Morroni G (2018). Characterization of *poxtA*, a novel phenicol-oxazolidinone-tetracycline resistance gene from an MRSA of clinical origin. J Antimicrob Chemother.

[15] Long KS, Poehlsgaard J, Kehrenberg C, Schwarz S, Vester B (2006). The Cfr rRNA methyltransferase confers resistance to phenicols, lincosamides, oxazolidinones, pleuromutilins, and streptogramin A antibiotics. Antimicrob Agents Chemother.

[16] Sato T (2018). Studies on molecular epidemiology of methicillinresistant *staphylococcus aureus* originated from livestock animals, meat products, and humans. Rakuno gakuen university.

[17] Nickoloff JA (1995). Electroporation protocols for microorganisms. Methods Mol Biol.

[18] Clinical and Laboratory Standard Institute

[19] Chen Z, Xiong Y, Tang Y, Zhao Y, Chen J (2022). *In vitro* activities of thiazolidione derivatives combined with daptomycin against clinical *Enterococcus faecium* strains. BMC Microbiol.

[20] Liu Z, Zhang H, Xiao X, Liu Y, Li R (2022). Comparison of fitness cost, stability, and conjugation frequencies of tet(X4)-positive plasmids in chicken and pig *Escherichia coli*. Antibiotics.

[21] Starikova I, Al-Haroni M, Werner G, Roberts AP, Sørum V (2013). Fitness costs of various mobile genetic elements in *Enterococcus faecium* and *Enterococcus faecalis*. J Antimicrob Chemother.

[22] Shen W, Cai C, Dong N, Chen J, Zhang R (2024). Mapping the widespread distribution and transmission dynamics of linezolid resistance in humans, animals, and the environment. Microbiome.

[23] Gawryszewska I, Żabicka D, Hryniewicz W, Sadowy E (2017). Linezolid-resistant enterococci in Polish hospitals: species, clonality and determinants of linezolid resistance. Eur J Clin Microbiol Infect Dis.

[24] Fujiya Y, Harada T, Sugawara Y, Akeda Y, Yasuda M (2021). Transmission dynamics of a linear vanA-plasmid during a nosocomial multiclonal outbreak of vancomycin-resistant enterococci in a non-endemic area, Japan. Sci Rep.

[25] Dorado-Morales P, Garcillán-Barcia MP, Lasa I, Solano C (2021). Fitness cost evolution of natural plasmids of *Staphylococcus aureus*. mBio.

[26] Hashimoto Y, Suzuki M, Kobayashi S, Hirahara Y, Kurushima J (2023). Enterococcal linear plasmids adapt to *Enterococcus faecium* and spread within multidrug-resistant clades. Antimicrob Agents Chemother.

[27] Lee T, Pang S, Abraham S, Coombs GW (2019). Antimicrobial-resistant CC17 *Enterococcus faecium*: the past, the present and the future. J Glob Antimicrob Resist.

[28] Lee JB, Lim JH, Park JH, Lee GY, Park KT (2024). Genetic characteristics and antimicrobial resistance of *Staphylococcus aureus* isolates from pig farms in Korea: emergence of cfr-positive CC398 lineage. BMC Vet Res.

